# Supplementary material for: Prior Influenza Infection Mitigates SARS-CoV-2 Disease in Syrian Hamsters
Source: Viruses. 2024 Feb 3;16(2):246. doi: 10.3390/v16020246 (PMC10891789; doi:10.3390/v16020246)
Supplement: Supplementary file 1 [file viruses-16-00246-s001.zip › Supp Tables and Figures.pdf]

Table S1: Histopathologic scoring system (lung)

The following data were collected from two longitudinal sections of the entire left lung in each animal

<u>Total histopathology score (Sum of subtotals 1-4)</u>

1. Nodular SARS2 bronchointerstitial inflammation (Subtotal 1)

a. Number of discrete foci bronchointerstitial inflammation

b. Destruction of alveolar walls (0 =no; 1 =yes)

c. Intra-alveolar hemorrhage (0=no; 1 =yes)

d. Percent alveolar consolidation (None =0; <5% = 1; 5-10% = 2; 10-20% = 3; 20-30% = 4; 30-40% = 5; 40-50% = 6; >50% = 7)

e. Alveolar/bronchiolar epithelial hyperplasia (None =0; 1-5 regions=1; > 5 regions = 2)

2. Diffuse H1N1 type interstitial pneumonia (Subtotal 2)

a. Percent of section affected by interstitial pneumonia (congestion, intraalveolar erythrocytes, intraalveolar edema, interstitial hypercellularity)

(None =0; <5% = 1; 5-10% = 2; 10-20% = 3; 20-30% = 4; 30-40% = 5; 40-50% = 6; >50% = 7)

3. Airway pathology (Subtotal 3)

a. Tracheal proprial inflammation: mononuclear (No=0; Yes =1)

b. Teracheal proprial inflammation: neutrophilic (No=0; Yes =1)

c. Tracheal epithelial necrosis (No=0; Yes =1)

d. Tracheal epithelial hyperplasia (No=0; Yes =1)

e. Bronchiolar epithelial necrosis/intrabronchial debris (0= none; 1=mild, 2= marked)

f. Peribronchiolar inflammatory infiltrate (0= none; 1=mild, 2= marked)

4. Vascular pathology (Subtotal 4)

a. Number of large to midsize vessels affected

b. Endothelial hypertrophy and elevation (None =0; 1-3 vessels affected =1; >3 vessels affected = 2)

c. Leukocyte adhesion (None =0; 1-3 vessels affected =1; >3 vessels affected = 2)

d. Leukocyte transmigration/vasculitis (None =0; 1-3 vessels affected =1; >3 vessels affected = 2)

e. Perivascular infiltrate (None =0; 1-3 vessels affected =1; >3 vessels affected = 2)

f. Number of vessels with thrombosis

Table S2. Primary antibodies for hamster SARS-CoV-2 and Influenza experiments

**Target**	**Primary antibody**	**Host**	**Concentration**	**Utility**
**Primary antibodies**				
Influenza A H1N1 (USSR)	ab20841 (Abcam)	Goat	1:400	IHC-P
SARS-CoV-2 NP	Cat # 40143-MM05 (Sino-Biological)	Mouse	1:100	IHC-P
CD3 clone SP7	Cat # ab16669 (Abcam)	Rabbit anti mouse/human	1:100	IHC-P
Aif-1 (Iba-1)	ACR290 (BioCare cat # 902-290-022619)	Rabbit anti mouse/human	1:100<br>1:300	IHC-P<br>Flow cytometry
MHC-II I-Ek clone 14-4-4S	AlexaFluor 647 (Biolegend cat # 110211)	Mouse anti-Rat	1:150	Flow cytometry
Immunoglobulin (H+L)	Cat # 6061 (Southern Biotech)	Goat Anti-Hamster	1:100	Flow cytometry
**Secondary antibodies**				
MACH 2 Mouse HRP-Polymer	Cat # MHRP520G (Biocare Medical)	Goat anti-mouse	Prediluted	IHC-P
MACH 2 Rabbit HRP-Polymer	Cat # RHRP520G (Biocare Medical)	Goat anti-Rabbit	Prediluted	IHC-P
Dylight488 (Excites at 493, Emits at 518)	Catalog # SA5-10166 (Thermo-Fisher)	Donkey anti-Mouse	1:100	IHC-P
DyLight549 (Excites at 556, Emits at 571)	Catalog # 35560 (Thermo-Fisher)	Goat anti-Rabbit	1:100	IHC-P

Rabbit IgG	Phycoerythrin (PE)<br>Cat# 4050-09S (Sthrn Biotech)	Goat anti-Rabbit	1:600	Flow cytometry

Table S3: Primer sequences for lung cytokine expression (qRT-PCR)

Gene	Forward primer (5' to 3')	Reverse Primer (5' to 3')	Reference
pH1N1 M gene	CTTCTAACCGAGGTCGAAACG	GGCATTTTGGACAAAKCGTCTA	[14]
SARS-CoV-2 RdRp	CGCATACAGTCTTRCAGGCT	GTGTGATGTTGAWATGACATGGTC	[14]
β-actin	ATGGCCAGGTCATCACCATTG	CAGGAAGGAAGGCTGGAAAAG	[14]
TNF-α	CACCCACCGTCAAGGATTCA	TTGGCTGGGCAATGAAGAGT	[14]
IL-6	TGTCTTCTTGGGACTGCTGC	CCAAACCTCCGACTTGTTGA	[14]
IFN-α	AGACTGGGAGTTGCCTGTGA	GAGGAATCCAGGGCTTTCCAG	[38]
IFN-γ	TGCATCTTGGCTTTGTTGCTC	TCCCCTCCATTCACGACATC	[14]
Mx-2	CCAGTAATGTGGACATTGCC	CATCAACGACCTTGTCTTCAGTA	[39]
Ace-2 p1	TGCAATGGTGAATCAGGGC	AGTCAGCATGGAGTTTTCCCA	NCBI Gene ID: 101823817
Ccl-2 (MCP-1) p1	CGGTCTCTGCAAGAACGCTT	GGGGAGTTAACGGAGTCTGG	NCBI Gene ID: 101839392
Ccl-8 (MCP2) p1	GATCTCCGCCATGCTTCTGT	ATGGGTGACTCATCTGGCTC	NCBI Gene ID: 101824449

14. Zhang AJ, Lee AC, Chan JF, Liu F, Li C, Chen Y, et al. Coinfection by Severe Acute Respiratory Syndrome Coronavirus 2 and Influenza A(H1N1)pdm09 Virus Enhances the Severity of Pneumonia in Golden Syrian Hamsters. Clin Infect Dis. 2021;72(12):e978-e92. Epub 2020/11/21. doi: 10.1093/cid/ciaa1747. PubMed PMID: 33216851; PubMed Central PMCID: PMCPMC7717201.
38. Bluyssen, H. A.; Nakamura, N.; Vlietstra, R. J.; Smit, E. M.; Hagemeijer, A.; Trapman, J., Isolation, properties and chromosomal localization of four closely linked hamster interferon-alpha-encoding genes. *Gene* **1995,** 158, (2), 295-300.

39. Zivcec, M.; Safronetz, D.; Haddock, E.; Feldmann, H.; Ebihara, H., Validation of assays to monitor immune responses in the Syrian golden hamster (Mesocricetus auratus). *J Immunol Methods* **2011,** 368, (1-2), 24-35.

Table S4: Descriptive data and unadjusted p-values for pair-wise comparisons of infection groups for total histopathology scores, flow cytometry data, viral load and cytokine/chemokine expression.

**A. Body weight and respiratory rate (H1N1, SARS-CoV-2, 3 hour dual, 48 hour dual infections)**										
	**1dpi**	**2dpi**	**3dpi**	**4dpi**	**5dpi**	**6dpi**	**7dpi**	**8dpi**	**9dpi**	**10dpi**
**Body weight comparisons** (pval)										
H1N1 vs Control	0.954	0.578	0.775	0.129	0.322	0.164	0.614	0.738	0.671	0.733
SARS-CoV-2 vs Control	0.615	**<.001**	**<.001**	**<.001**	**<.001**	**<.001**	**<.001**	**<.001**	**<.001**	**<.001**
Dual 3hrs vs SARS-CoV-2	0.958	0.822	0.498	0.837	0.388	**0.074**	**0.002**	**0.013**	**0.036**	**0.007**
Dual 48hrs vs SARS-CoV-2	0.556	**0.030**	**0.022**	0.134	**<.001**	**<.001**	**<.001**	**<.001**	**<.001**	**<.001**
**Respiratory rate comparisons** (pval)										
H1N1 vs Control	0.168	0.206	0.469	0.433	0.979	0.962	0.325	0.853	0.064	0.234
SARS-CoV-2 vs Control	0.319	0.447	0.968	**0.011**	**<.001**	**<.001**	**<.001**	0.814	0.729	**0.046**
Dual 3hrs vs SARS-CoV-2	0.730	0.273	0.346	**0.029**	0.476	0.529	0.380	**0.004**	0.227	0.602
Dual 48hrs vs SARS-CoV-2	0.735	0.372	0.954	**0.034**	0.100	**0.210**	0.065	0.925	0.865	0.182

**B. Lung pathology and flow cytometry (H1N1, SARS-CoV-2, 3 hour dual, 48 hour dual infections)**				
	**2 dpi**	**4 dpi**	**7 dpi**	**10 dpi**
**Total histopathology severity score** (mean, sd)				
H1N1	8.33 (2.25)	9.83 (2.79)	5.67 (2.42)	1.67 (1.03)
SARS-CoV-2	3.5 (3.21)	13.67 (6.09)	27.5 (5.24)	12.33 (4.93)
Dual 3 hours	7.83 (2.14)	16.00 (5.76)	34.67 (9.35)	17.50 (11.17)
Dual 48 hours	10.83 (6.88)	10.33 (7.06)	16.67 (5.50)	2.00 (1.55)
**Comparisons** (pval)				
H1N1 vs SARS-CoV-2	**0.032**	0.086	**<0.001**	**<0.001**
Dual 3hrs vs SARS-CoV-2	0.230	0.515	0.050	0.154
Dual 48hrs vs SARS-CoV-2	**0.021**	0.282	**0.001**	**0.002**
**% Granulocytes, BAL** (mean, sd)				
H1N1	0.53 (0.23)	0.54 (0.26)	0.43 (0.30)	0.70 (0.25)

SARS-CoV-2	0.47 (0.13)	0.45 (0.10)	0.22 (0.04)	0.57 (0.10)
Dual 3 hours	0.27 (0.08)	0.15 (0.07)	0.14 (0.04)	0.26 (0.05)
Dual 48 hours	0.31 (0.19)	0.37 (0.09)	0.35 (0.05)	0.65 (0.07)
Control	0.78 (0.11)	0.51 (0.29)	0.59 (0.25)	0.86 (0.04)
**Comparisons** (pval)				
H1N1 vs Control	0.691	0.158	**0.006**	0.978
SARS-CoV-2 vs Control	0.338	**0.019**	**<0.0001**	0.163
Dual 3hrs vs SARS-CoV-2	0.056	**0.005**	0.401	**0.003**
Dual 48hrs vs SARS-CoV-2	0.127	0.450	0.219	0.384
**% Granulocytes, blood** (mean, sd)				
H1N1	0.29 (0.06)	0.29 (0.09)	0.21 (0.06)	0.22 (0.05)
SARS-CoV-2	0.48 (0.11)	0.31 (0.08)	0.29 (0.07)	0.23 (0.01)
Dual 3 hours	0.45 (0.14)	0.40 (0.13)	0.25 (0.06)	0.31 (0.19)
Dual 48 hours	0.36 (0.09)	0.39 (0.04)	0.26 (0.06)	0.29 (0.06)
Control	0.18 (0.08)	0.25 (0.09)	0.27 (0.03)	0.29 (0.09)
**Comparisons** (pval)				
H1N1 vs Control	0.643	0.236	0.438	0.643
SARS-CoV-2 vs Control	**<0.0001**	0.134	0.550	0.154
Dual 3hrs vs SARS-CoV-2	0.619	0.081	0.535	0.619
Dual 48hrs vs SARS-CoV-2	**0.039**	0.129	0.609	**0.039**
**Fraction APC Aif1+ Iek+, BALF** (mean, sd)				
H1N1	0.15 (0.09)	0.09 (0.03)	0.15 (0.08)	0.07 (0.05)
SARS-CoV-2	0.15 (0.08)	0.10 (0.05)	0.07 (0.01)	0.04 (0.02)
Dual 3 hours	0.37 (0.12)	0.34 (0.15)	0.29 (0.01)	0.38 (0.02)
Dual 48 hours	0.24 (0.11)	0.11 (0.02)	0.07 (0.01)	0.06 (0.02)
Control	0.07 (0.06)	0.12 (0.17)	0.13 (0.18)	0.12 (0.14)
**Comparisons** (pval)				
H1N1 vs Control	0.691	0.158	**0.006**	0.978
SARS-CoV-2 vs Control	0.338	**0.019**	**<0.0001**	0.163
Dual 3hrs vs SARS-CoV-2	0.056	**0.005**	0.401	**0.003**

Dual 48hrs vs SARS-CoV-2	0.127	0.450	0.219	0.384
**Fraction APC Aif1+ Iek+, blood** (mean, sd)				
H1N1	0.08 (0.03)	0.06 (0.02)	0.05 (0.03)	0.05 (0.01)
SARS-CoV-2	0.06 (0.03)	0.05 (0.01)	0.05 (0.01)	0.06 (0.01)
Dual 3 hours	0.05 (0.02)	0.05 (0.02)	0.05 (0.01)	0.08 (0.03)
Dual 48 hours	0.06 (0.04)	0.04 (0.02)	0.04 (0.01)	0.05 (0.01)
Control	0.07 (0.01)	0.06 (0.02)	0.06 (0.02)	0.06 (0.02)
**Comparisons** (pval)				
H1N1 vs Control	0.493	0.619	0.844	0.322
SARS-CoV-2 vs Control	0.561	0.305	0.729	0.800
Dual 3hrs vs SARS-CoV-2	0.572	0.885	0.997	0.209
Dual 48hrs vs SARS-CoV-2	0.609	0.391	0.223	0.382
**Fraction APC Aif1- Iek+, BALF** (mean, sd)				
H1N1	0.11 (0.09)	0.08 (0.06)	0.07 (0.05)	0.07 (0.08)
SARS-CoV-2	0.06 (0.02)	0.06 (0.02)	0.12 (0.05)	0.05 (0.01)
Dual 3 hours	0.06 (0.04)	0.07 (0.07)	0.13 (0.07)	0.06 (0.01)
Dual 48 hours	0.17 (0.07)	0.09 (0.03)	0.15 (0.02)	0.06 (0.02)
Control	0.13 (0.17)	0.05 (0.07)	0.05 (0.05)	0.05 (0.03)
**Comparisons** (pval)				
H1N1 vs Control	0.296	0.432	0.543	0.605
SARS-CoV-2 vs Control	**0.012**	0.829	0.063	0.974
Dual 3hrs vs SARS-CoV-2	0.933	0.761	0.746	0.779
Dual 48hrs vs SARS-CoV-2	**0.003**	0.382	0.435	0.877
**Fraction APC Aif1- Iek+, blood** (mean, sd)				
H1N1	0.05 (0.01)	0.07 (0.01)	0.07 (0.01)	0.08 (0.03)
SARS-CoV-2	0.05 (0.01)	0.06 (0.01)	0.05 (0.02)	0.05 (0.01)
Dual 3 hours	0.07 (0.03)	0.07 (0.01)	0.09 (0.03)	0.05 (0.02)
Dual 48 hours	0.07 (0.01)	0.08 (0.01)	0.09 (0.03)	0.08 (0.01)
Control	0.08 (0.02)	0.10 (0.02)	0.08 (0.03)	0.09 (0.01)
**Comparisons** (pval)				

H1N1 vs Control	0.058	**0.033**	**0.032**	0.343
SARS-CoV-2 vs Control	**0.021**	**0.004**	**<0.0001**	**0.004**
Dual 3hrs vs SARS-CoV-2	**0.025**	0.246	**0.001**	0.794
Dual 48hrs vs SARS-CoV-2	0.068	0.165	**<0.0001**	**0.042**
**Fraction APC Aif1 + Iek-, BALF** (mean, sd)				
H1N1	0.011 (0.01)	0.018 (0.02)	0.022 (0.01)	0.006 (0.00)
SARS-CoV-2	0.009 (0.00)	0.011 (0.00)	0.02 1(0.00)	0.007 (0.00)
Dual 3 hours	0.024 (0.02)	0.006 (0.00)	0.007 (0.00)	0.007 (0.00)
Dual 48 hours	0.005 (0.00)	0.010 (0.00)	0.004 (0.00)	0.004 (0.00)
Control	0.017 (0.01)	0.007 (0.01)	0.018 (0.02)	0.006 (0.00)
**Comparisons** (pval)				
H1N1 vs Control	0.885	0.165	0.199	0.212
SARS-CoV-2 vs Control	0.917	0.813	0.237	0.237
Dual 3hrs vs SARS-CoV-2	**0.019**	0.494	**0.029**	1.000
Dual 48hrs vs SARS-CoV-2	0.510	0.854	0.080	0.635
**Fraction APC Aif1 + Iek-, blood** (mean, sd)				
H1N1	0.06 (0.02)	0.06 (0.01)	0.06 (0.01)	0.05 (0.01)
SARS-CoV-2	0.04 (0.03)	0.04 (0.02)	0.07 (0.01)	0.06 (0.02)
Dual 3 hours	0.04 (0.01)	0.04 (0.01)	0.04 (0.01)	0.05 (0.02)
Dual 48 hours	0.04 (0.01)	0.04 (0.01)	0.04 (0.01)	0.03 (0.01)
Control	0.06 (0.02)	0.04 (0.01)	0.05 (0.01)	0.04 (0.01)
**Comparisons** (pval)				
H1N1 vs Control	0.490	0.264	0.054	0.572
SARS-CoV-2 vs Control	**0.047**	0.332	**0.005**	**0.049**
Dual 3hrs vs SARS-CoV-2	0.585	0.649	**0.023**	0.131
Dual 48hrs vs SARS-CoV-2	0.982	0.817	**0.002**	**0.006**

C. Nasal viral gene expression (H1N1, SARS-CoV-2, 48 hour dual infection)

	1dpi	2dpi
**NASAL H1N1 M gene fold change** (mean, sd)		
H1N1	9435.80 (8036.30)	37026.07 (6849.41)

SARS-CoV-2	n/a	n/a
Dual 48 hours	19782.53 (8954.46)	8221.51 (3729.28)
**Comparisons** (pval)		
H1N1 Day1 vs Day 2	**0.0106**	
Dual 48 hours Day 1 vs Day 2	0.1079	
Dual 48 hours vs H1N1	0.2106	**0.0031**
**NASAL SARS-CoV-2 RdRp fold change** (mean, sd)		
H1N1	n/a	n/a
SARS-CoV-2	15352.48 (24580.58)	22599.48 (15558.39)
Dual 48 hours	21427.22 (4567.65)	13288.91 (2964.20)
**Comparisons** (pval)		
SARS-CoV-2 Day1 vs Day 2	0.6884	
Dual 48 hours Day 1 vs Day 2	0.0608	
Dual 48 hours vs SARS-CoV-2	0.6955	0.3662

D. Lung viral gene and cytokine expression (H1N1, SARS-CoV-2, 48 hour dual infection)

	**2 dpi**	**4 dpi**	**7 dpi**	**10 dpi**
**LUNG H1N1 M gene fold change** (mean, sd)				
H1N1	3370.79 (4943.67)	17185.87(36708.68)	4667.20 (11067.09)	43.01(72.63)
SARS-CoV-2	n/a	n/a	n/a	n/a
Dual 48 hours	18284.55 (26308.11)	1516.72	12.80	20.05
Comparisons (pval)				
H1N1 vs SARS-CoV-2	n/a	n/a	n/a	n/a
Dual 48 hours vs H1N1	0.675	0.863	0.267	0.770
**LUNG SARS-CoV-2 RdRp fold change** (mean, sd)				
H1N1	n/a	n/a	n/a	n/a
SARS-CoV-2	250598.27 (330879.65)	356412.22 (349772.07)	1253.33 (2493.64)	14.05 (8.24)
Dual 48 hours	6534.47 (8055.98)	92637.49 (86960.68)	257.12 (166.70)	15.13 (20.72)
**Comparisons** (pval)				
H1N1 vs SARS-CoV-2	n/a	n/a	n/a	n/a

Dual 48 hours vs SARS-CoV-2	**<0.001**	0.080	0.551	0.671
**LUNG IL-6 fold change (mean, sd)**				
H1N1	0.84 (0.12)	1.71 (1.68)	2.87 (2.10)	2.31 (2.58)
SARS-CoV-2	2.60 (1.76)	16.32 (12.61)	3.35 (2.17)	1.11 (0.40)
Dual 48 hours	3.31 (3.25)	4.02 (2.26)	1.74 (0.54)	1.24 (0.40)
**Comparisons** (pval)				
H1N1 vs SARS-CoV-2	0.565	**<0.001**	0.787	0.636
Dual 48 hours vs SARS-CoV-2	0.747	**<0.001**	0.514	0.962
**LUNG Ccl2 fold change** (mean, sd)				
H1N1	2.71 (1.24)	5.00 (7.82)	12.22 (12.13)	2.51 (1.12)
SARS-CoV-2	3.32 (2.21)	66.03 (43.51)	41.07 (19.09)	2.54 (0.37)
Dual 48 hours	11.98 (15.55)	27.25 (11.22)	21.83 (9.67)	2.91 (0.78)
**Comparisons** (pval)				
H1N1 vs SARS-CoV-2	0.996	**<0.001**	**0.005**	0.997
Dual 48 hours vs SARS-CoV-2	0.356	**0.001**	0.057	0.970
**LUNG CxCl10 fold change** (mean, sd)				
H1N1	2.05 (1.25)	4.38 (5.97)	6.81 (5.55)	1.99 (0.59)
SARS-CoV-2	18.48 (25.19)	84.90 (75.72)	13.60 (6.30)	2.26 (0.63)
Dual 48 hours	17.78 (22.52)	37.18 (17.75)	10.18 (4.81)	2.63 (0.7)
**Comparisons** (pval)				
H1N1 vs SARS-CoV-2	0.310	**<0.001**	0.673	0.987
Dual 48 hours vs SARS-CoV-2	0.968	**0.008**	0.842	0.983
**LUNG Ccl8 fold change** (mean, sd)				
H1N1	1.19 (0.27)	1.71 (0.59)	5.04 (4.37)	1.64 (0.58)
SARS-CoV-2	3.65 (3.64)	8.8 (8.4)	7.09 (1.71)	1.05 (0.11)
Dual 48 hours	4.18 (4.22)	6.99 (3.38)	6.28 (2.87)	1.45 (0.33)
**Comparisons** (pval)				
H1N1 vs SARS-CoV-2	0.252	**0.002**	0.338	0.782
Dual 48 hours vs SARS-CoV-2	0.813	0.421	0.720	0.859
**LUNG Interferon-gamma fold change** (mean, sd)				

H1N1	0.77 (0.34)	6.69 (7.79)	4.24 (2.33)	6.64 (11.77)
SARS-CoV-2	3.51 (3.10)	28.74 (20.42)	6.32 (1.78)	2.54 (0.37)
Dual 48 hours	7.72 (10.42)	16.73 (5.60)	6.36 (2.70)	2.17 (1.07)
**Comparisons** (pval)				
H1N1 vs SARS-CoV-2	0.650	**<0.001**	0.692	0.436
Dual 48 hours vs SARS-CoV-2	0.404	**0.021**	0.994	0.942
**LUNG Interferon- α fold change** (mean, sd)				
H1N1	0.83 (0.27)	1.06 (0.35)	1.63 (0.47)	0.96 (0.39)
SARS-CoV-2	0.71 (0.26)	1.54 (0.72)	0.77 (0.43)	0.74 (0.51)
Dual 48 hours	0.77 (0.27)	0.85 (0.13)	0.62 (0.09)	0.84 (0.10)
**Comparisons** (pval)				
H1N1 vs SARS-CoV-2	0.230	**0.008**	0.096	0.551
Dual 48 hours vs SARS-CoV-2	0.081	**0.017**	**0.020**	0.202
**LUNG Ace2 fold change** (mean, sd)				
H1N1	0.77 (0.29)	0.97 (0.24)	1.51 (1.06)	1.48 (0.54)
SARS-CoV-2	2.02 (2.02)	0.75 (0.45)	0.60 (0.24)	1.49 (0.51)
Dual 48 hours	0.86 (0.25)	0.93 (0.50)	1.06 (0.30)	1.29 (0.18)
**Comparisons** (pval)				
H1N1 vs SARS-CoV-2	**0.017**	0.666	0.078	0.978
Dual 48 hours vs SARS-CoV-2	**0.010**	0.673	0.296	0.640
**LUNG Mx-2 fold change** (mean, sd)				
H1N1	19.48 (3.54)	4.80 (3.99)	3.82 (2.65)	1.48 (0.58)
SARS-CoV-2	55.30 (67.91)	25.82 (13.23)	12.60 (5.54)	1.51 (0.26)
Dual 48 hours	16.66 (9.33)	20.95 (10.42)	16.74 (10.39)	1.95 (0.34)
**Comparisons** (pval)				
H1N1 vs SARS-CoV-2	**0.016**	0.149	0.542	0.999
Dual 48hrs vs SARS-CoV-2	**0.012**	0.742	0.780	0.976
**LUNG TNF-α fold change** (mean, sd)				
H1N1	1.01 (0.54)	1.85 (0.32)	2.4 (1.2)	2.97 (3.88)
SARS-CoV-2	1.13 (0.55)	1.24 (0.53)	0.73 (0.19)	1.23 (0.47)

Dual 48 hours	1.09 (0.24)	1.29 (0.55)	0.49 (0.13)	0.91 (0.44)
**Comparisons** (pval)				
H1N1 vs SARS-CoV-2	0.952	0.481	0.083	0.053
Dual 48hrs vs SARS-CoV-2	0.901	0.843	0.332	0.216

Cytokine and viral load expression was expressed as fold difference of infected compared to control samples. N=24/infection group (split by sex, sections A, B, D), n=3 per group (males, section C). Sexes are combined. Unadjusted p-values for pair-wise comparisons of infection groups for total histopathology scores, viral load, flow cytometry data and cytokine/chemokine expression. Bold: $p<0.05$.

Table S5: Unadjusted p-values for sex effect on body weight, respiratory weight, total histopathology score, viral load, cytokine/chemokine expression and immune cell populations across infection groups

Infection group	p-val
**A. Clinical and histopathologic sex differences**	
**Body weight**	
Control	0.293
H1N1 only	**0.002**
SARS2 only	**0.025**
Dual 3hrs	< **0.001**
Dual 48hrs	**0.008**
**Respiratory rate**	
Control	0.799
H1N1 only	0.090
SARS2 only	**0.008**
Dual 3hrs	**0.002**
Dual 48hrs	0.564
**Total histopathology score**	
H1N1 only	0.982
SARS2 only	0.301
Dual 3hrs	0.127
Dual 48hrs	0.910
**B. Sex differences in viral load and cytokine expression (lung)**	
**H1N1 M gene**	
H1N1 only	0.756

SARS2 only	n/a
Dual 3hrs	0.836
Dual 48hrs	0.770
**SARS2 RdRp**	
H1N1 only	n/a
SARS2 only	0.674
Dual 3hrs	0.928
Dual 48hrs	0.899
**IL-6**	
H1N1 only	0.762
SARS2 only	**0.009**
Dual 3hrs	0.628
Dual 48hrs	0.725
**Ccl2**	
H1N1 only	0.839
SARS2 only	0.087
H1N1<SARS2 3hrs	0.159
H1N1<SARS2 48hrs	0.699
**CxCl10**	
H1N1 only	0.973
SARS2 only	**0.020**
Dual 3hrs	0.458
Dual 48hrs	0.889
**Ccl8**	
H1N1 only	0.742
SARS2 only	0.106
Dual 3hrs	0.206

Dual 48hrs	0.445
**Interferon-gamma**	
H1N1 only	0.981
SARS2 only	0.340
Dual 3hrs	0.499
Dual 48hrs	0.898
**Interferon-alpha**	
H1N1 only	0.737
SARS2 only	**0.023**
Dual 3hrs	0.983
Dual 48hrs	0.820
**Ace-2**	
H1N1 only	0.610
SARS2 only	**0.006**
Dual 3hrs	0.560
Dual 48hrs	0.238
**Mx-2**	
H1N1 only	0.995
SARS2 only	0.166
Dual 3hrs	0.481
Dual 48hrs	0.947
**TNF-α**	
H1N1 only	0.747
SARS2 only	0.962
Dual 3hrs	0.514
Dual 48hrs	**<0.001**

**C. Sex differences in immune cell populations (BALF and blood)**		
**% granulocytes of leukocytes**	BALF	Blood
Control	0.525	0.621
H1N1 only	0.455	0.671
SARS2 only	0.651	0.086
Dual 3hrs	0.440	0.609
Dual 48hrs	0.405	0.911
**% Aif1+ IEk- myeloid of leukocytes**	BALF	Blood
Control	0.471	**0.019**
H1N1 only	0.960	0.440
SARS2 only	0.819	**0.027**
Dual 3hrs	0.110	0.783
Dual 48hrs	0.773	0.440
**% Aif1- IEk+ myeloid of leukocytes**	BALF	Blood
Control	0.201	0.992
H1N1 only	0.881	0.738
SARS2 only	0.964	0.378
Dual 3hrs	0.421	0.860
Dual 48hrs	0.600	0.288
**% Aif1+ IEk+ myeloid of leukocytes**	BALF	Blood
Control	0.052	0.728
H1N1 only	0.913	0.919
SARS2 only	0.552	0.060
Dual 3hrs	0.169	0.177
Dual 48hrs	0.620	0.002

To analyze outcomes by sex, models were computed that included infection group, sex, and an infection group by sex interaction with correlations from repeated measurement across time accounting for body weight and respiration. The dataset was not sufficiently powered to perform these analyses by individual day post infection.

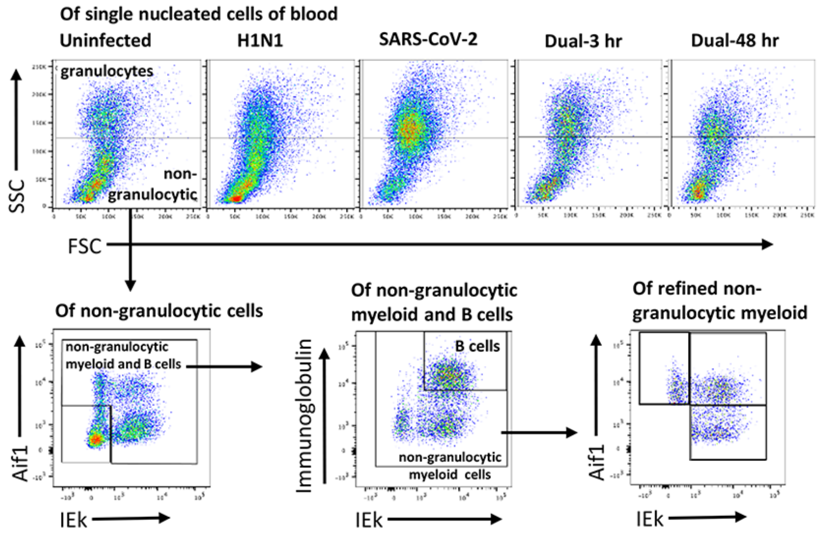

Figure S1: Summary of the gating strategy for cell subset analysis of flow cytometry data. Shown are representative flow cytometry plots of blood obtained from hamsters at 2 dpi. Granulocytic cells, known to be primarily neutrophils, are distinguished by their SSC and FSC properties. Due to the unfortunate paucity of validated reagents for the phenotyping of hamster cells, identification of non-granulocytic cells relied on expression levels of MHC II (IEk), Aif1 (aka Iba1) and surface immunoglobulin (Ig) levels, an approach that has proved helpful with other species with few specific validated antibodies. To further subset non-granulocytic myeloid cells, lymphocyte populations were gated to exclude Aif1- IEk- cells (T lymphocytes, NK cells and other innate lymphoid cell types) and B cells (IEk+ Ig++). The remaining myeloid cells were then distinguished by their varied expression of Aif1 and IEk.

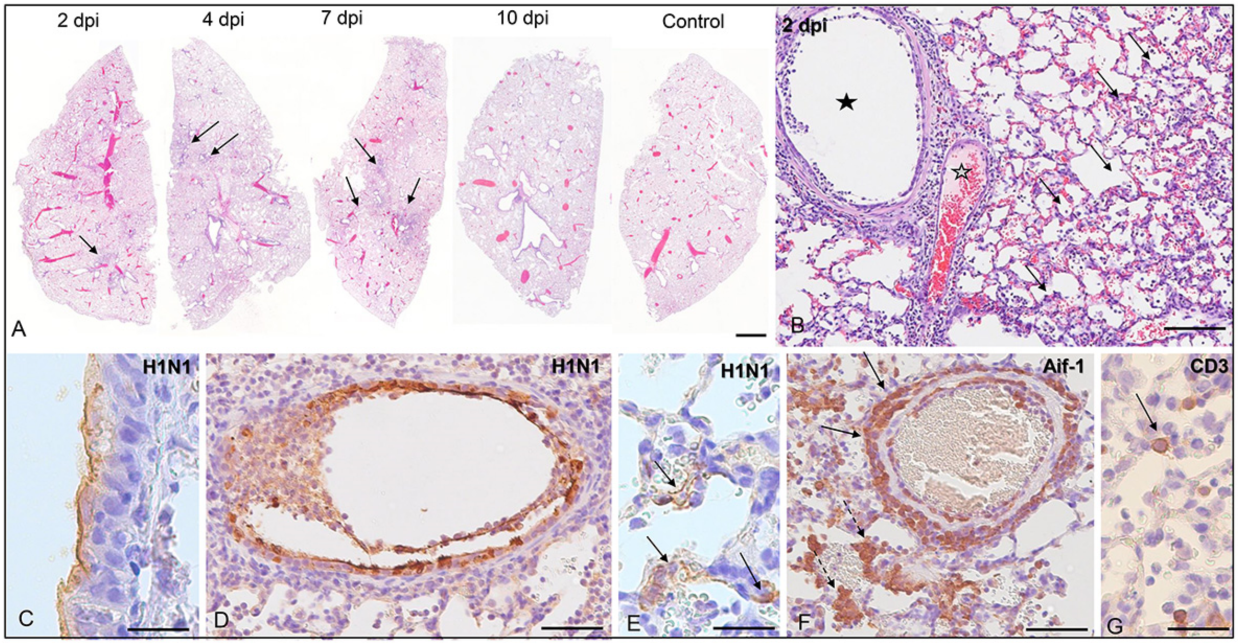

Figure S2: Histopathology, H1N1 only infection

A. Subgross histopathology of left lung, 2-10 dpi (H1N1 infected) and control. Regions of mild interstitial pneumonia are shown with arrows.

B. Characteristic lung histopathology, 2 dpi. Airway pathology (black asterisk; bronchiolar epithelial necrosis, peribronchiolar round cell inflammation), perivascular inflammation (grey asterisk) and parenchymal pathology (arrows; congestion, mild intra-alveolar and interstitial macrophage infiltration, and occasional intra-alveolar hemorrhage) are indicated.

C. Trachea, immunohistochemistry, H1N1, 2 dpi. H1N1 antigen is evident along apical surfaces of epithelial cells.

D. Bronchiole, immunohistochemistry, H1N1, 2 dpi. H1N1 antigen is detected in necrotic and intact bronchiolar epithelium.

E. Alveoli, immunohistochemistry, H1N1, 2 dpi. H1N1 antigen is detected in alveolar epithelium (arrows).

F. Pulmonary vein, immunohistochemistry, Aif-1, 4 dpi. Parenchymal inflammatory infiltrate is macrophage dominated (dashed arrows). Vessels demonstrate monocyte margination and perivascular accumulation (solid arrow).

G. Parenchyma, immunohistochemistry, CD3, 4 dpi. Individual lymphocytes are evident in parenchyma by 4 dpi (arrow). Bar = 1mm (A), 100μm (B), 50μm (D, F), 20μm (C, E, G).

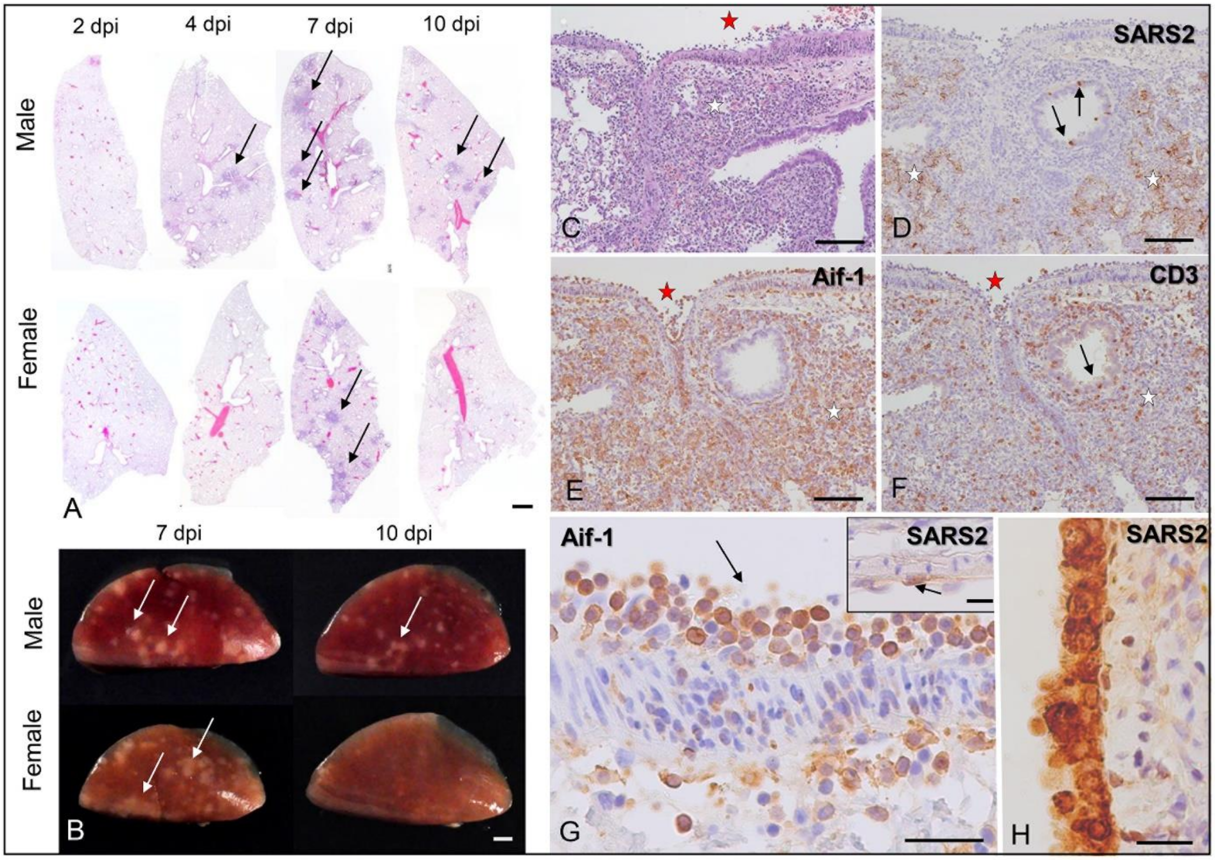

Figure S3: SARS-CoV-2 only phenotype

A. Representative samples, subgross pathology, left lung, males and females, 2-10 dpi. Nodular broncho-interstitial pneumonia (arrows) appears earlier in males (4 dpi) and takes longer to resolve.

B. Representative samples, gross pathology, left lung, males and females, 7- and 10- dpi. Nodular broncho-interstitial pneumonia manifests as discrete pale foci scattered randomly throughout the lung (arrows). Resolution in females is typically more advanced at 10 dpi than in males.

C. Lung histopathology, male, 4 dpi. Broncho-interstitial pneumonia is characterized by peribronchiolar and perivascular parenchymal inflammation, with intense vascular leucocyte margination and transmigration (red asterisk).

D. SARS2 immunohistochemistry, male, 4 dpi. SARS NP is detected in bronchiolar epithelium (arrows) and parenchyma, but not vasculature.

E. Aif-1 immunohistochemistry, male, 4 dpi. Parenchymal inflammatory infiltrate is dense, and macrophage dominated (white asterisk). Vessels are packed with adherent monocytes (red asterisk).

F. CD3 immunohistochemistry, male, 4 dpi. T lymphocytes are evident in bronchioles (arrow), parenchyma (white asterisk) and adherent to endothelium (red asterisk).

G. Vascular pathology, Aif-1 immunohistochemistry, male, 4 dpi. Intense monocytic margination (arrow), transmigration and perivascular macrophage accumulation is evident. Detection of SARS2 NP is rare within endothelial cells (inset, arrow).

H. Bronchiolus, SARS2 immunohistochemistry, male, 4 dpi. Diffuse expression of SARS2 NP is evident throughout cytoplasm of bronchiolar epithelium.

Bar = 1mm (A, B); 100 µm (C-F); 20 µm (G, H); 5µm (G, inset)

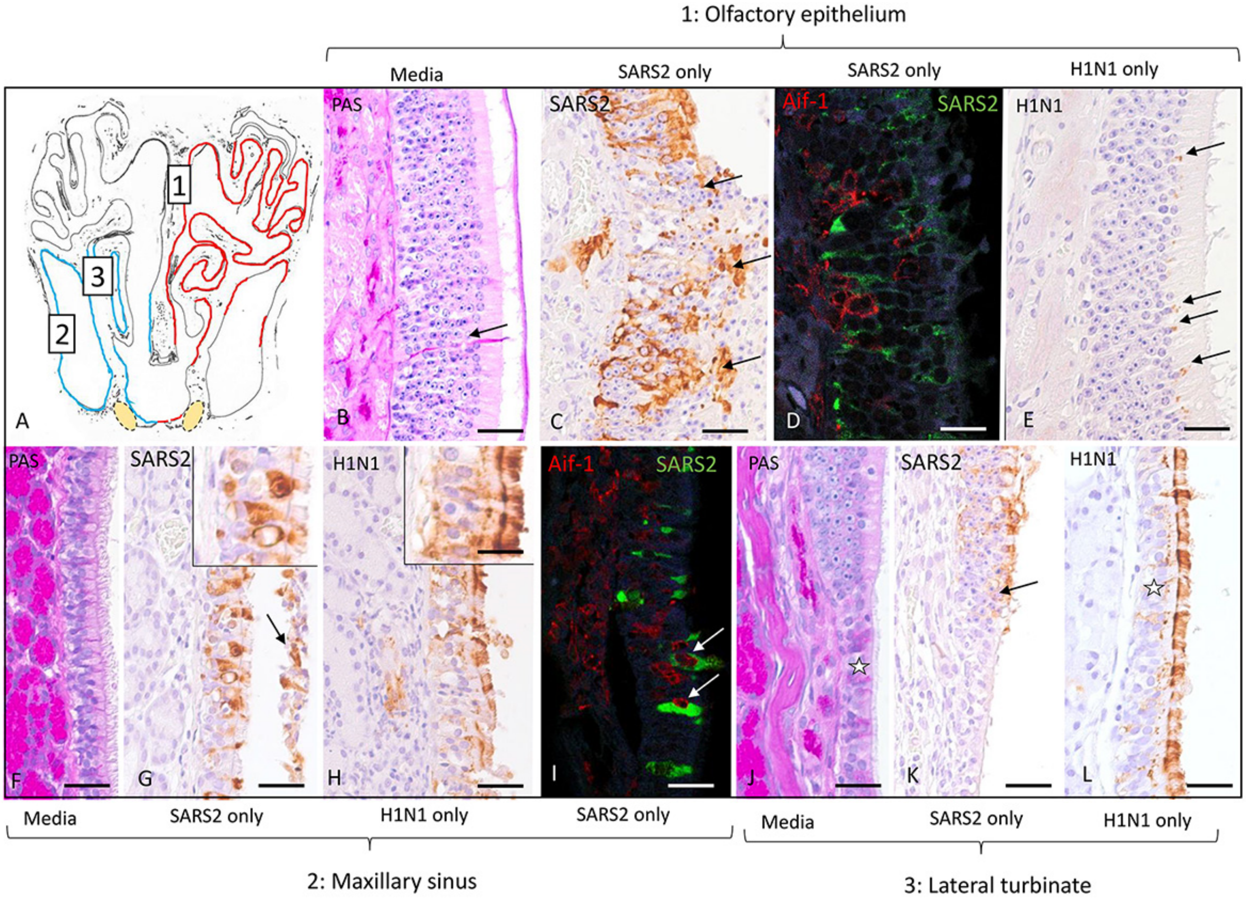

Figure S4: Nasal pathology and viral immunohistochemistry in H1N1 and SARS-CoV-2 only animals at 2 dpi.

A: Diagram of mid-nasal cross section. Viral distribution and pathology are illustrated in olfactory epithelium (1), respiratory epithelium of the maxillary sinus (2), and the junction olfactory and respiratory epithelium in lateral turbinates (3). Location of nasal associated lymphoid tissue (NALT) is indicated with yellow ovals.

B-E: Area 1, Olfactory epithelium: Normal olfactory epithelium (B, media control animal) with a duct delivering mucus from proprial glands indicated (arrow). SARS-CoV-2 only infected animal (C, D). The presence of SARS-CoV-2 NP throughout olfactory epithelium (C) is accompanied by widespread epithelial fragmentation (arrows) and vigorous macrophage

infiltration (D). H1N1 only infected animals present with paranuclear H1N1 immunostaining in rare olfactory epithelial cells (arrows), with no associated cytopathology.

F-I: Area 2, Respiratory epithelium in maxillary sinus. In SARS-CoV-2 only infected animals (G), SARS-CoV-2 NP is evident in cilia and throughout cell bodies (inset), accompanied by epithelial necrosis (arrow, G). In H1N1 only animals (H), robust H1NI immunostaining is evident in cilia and apical cell bodies (inset), with minimal associated cytopathology (H).

J-L: Area 3, Junction of olfactory and respiratory epithelium, lateral turbinates: Abrupt cessation of SARS-CoV-2 NP expression is noted as olfactory epithelium transitions to respiratory epithelium (arrow, K). Extensive H1N1 expression in cilia and apices of respiratory epithelium below the transition point (asterisk J, L).

Bar =10µm (I, J); 20 µm (remaining panels)
